# 557 Understanding Burns and Diabetes: A 12-Year Analysis of Outcomes and Challenges in Lower Extremity Reconstruction

**DOI:** 10.1093/jbcr/iraf019.186

**Published:** 2025-04-01

**Authors:** José Arellano, Christopher Fedor, Mare Kaulakis, Alexis Henderson, Hilary Liu, Garth Elias, Alain Corcos, Jenny Ziembicki, Francesco Egro

**Affiliations:** University of Pittsburgh Medical Center; University of Pittsburgh School of Medicine; University of Pittsburgh School of Medicine; University of Pittsburgh School of Medicine; University of Pittsburgh Medical Center; University of Pittsburgh Medical Center; University of Pittsburgh Medical Center; University of Pittsburgh Medical Center; University of Pittsburgh Medical Center

## Abstract

**Introduction:**

Burn injuries significantly contribute to accidental injuries and fatalities worldwide, affecting an estimated eight million people annually. At the same time, diabetes impacts approximately 422 million people globally, with the majority living in low- and middle-income countries, and is directly responsible for 1.5 million deaths each year. Both the number of cases and the prevalence of diabetes have been steadily increasing over the past few decades. As a chronic metabolic disease, diabetes is characterized by elevated blood glucose levels, which impair small blood vessels and hinder wound healing, posing unique challenges for burn management. This 12-year single-institution study examines the outcomes and complications in diabetic patients undergoing lower extremity burn reconstruction, exploring the impact of diabetes on burn recovery.

**Methods:**

A retrospective analysis was carried out on diabetic patients with lower extremity burns treated at a single ABA-verified burn center from 2012 to 2023. The data collected included demographics, burn characteristics, treatment methods, and outcomes. Logistic regression was used to examine the associations between burn-related factors and the probability of requiring surgical intervention.

**Results:**

A total of 571 patients were included in the analysis, 65.3% of whom were male. Among them, 100 patients (18.0%) had diabetes, while 454 (82.0%) did not. The overall surgery rate was 52.5%, with no significant difference between diabetic and non-diabetic patients (p=0.691). There were no significant differences in the risk of hypertrophic scarring (p=0.091), contracture formation (p=0.326), or graft loss (p=0.250). However, diabetic patients had a higher risk of reoperation (54.0% vs. 38.6%, p=0.004), osteomyelitis (5.8% vs. 0.48%, p< 0.001), and cellulitis (46.2% vs. 27.5%, p< 0.001). In multivariate analysis, adjusting for age and total body surface area (TBSA), diabetes was associated with twice the odds of re-intervention (OR=2.00, 95% CI [1.25, 3.20], p=0.004) and six times the odds of developing osteomyelitis (OR=6.04, 95% CI [1.10, 33.22], p=0.038). However, after adjustment, diabetes was no longer a significant predictor of cellulitis (OR=1.55, 95% CI [0.94, 2.55], p=0.084).

**Conclusions:**

Diabetic patients undergoing lower extremity burn reconstruction have significantly higher risks of reoperation and osteomyelitis, emphasizing the need for targeted surgical strategies and careful postoperative management. While diabetes does not increase scarring, contracture, or graft loss, its link to serious complications underscores the importance of early intervention.

**Applicability of Research to Practice:**

This research offers insights that can improve the management of lower extremity burns in diabetic patients. By identifying key factors affecting complications and surgical needs, it helps guide treatment strategies and optimize patient outcomes.

**Funding for the Study:**

N/A

